# Medical and Philosophical News

**Published:** 1781-02

**Authors:** 


### SECTION III.

### Medical and Philosophical News.

THE Society of Natural Hiftorians at Berlin have propofed the following queftion for a prize of twenty ducats: 1. "How "long is the virus producing hydrophobia ca"pable of remaining in an animal, and what is "the length of time required for communicat"ing it? 2. How long can this fame virus "remain in the body without manifefting itfelf? "From the moment the difeafe is communi"cated, what are the moft efficacious means of "obviating its effects?" The differtations on this fubject are to be written in French or Latin, and fent to M. Otho, fecretary to the Society at Berlin, on or before the 27th of December 1781.

---

Dr. Gilbert Blane, phyfician to the fleet in the Weft Indies, in a letter addreffed to Dr. Hunter, and read at the Royal Society, has given a very accurate account of the late hurricane; and has likewife attempted a new theory of of hurricane. Coldneſs and moiſture in tropical climates are generally productive of the moſt pernicious effects; but this ſtorm, it ſeems, was not more remarkable for its dreadful ravages while it laſted, than for the ſalubrity of its effects after it was over. The ſick in general were benefited by it. Patients labouring under pulmonary complaints were thoſe who were the moſt ſenſibly relieved by it. Several hectic fevers were greatly mitigated, and ſome few cured by it, at leaſt for a time. Amongſt other inſtances of its ſalutary operation, Dr. Blane ſpeaks of a lady who had been confined to her room for ten days with a pleuriſy, and who paſſed the night ſucceeding the hurricane in the open air, and on the wet ground, yet ſhe ſoon recovered.

---

M. de Villers, an ingenious naturaliſt at Lyons, has lately diſtributed propoſals for publiſhing by ſubſcription a work, intitled, "Les Inſectes de "la France decrits et claſſés ſelon la methode de "Linnæus." It will conſiſt of 4 vols. in 4to, and contain 200 engravings by the beſt artiſts, from drawings by M. Gonicbon. About 2500 inſects will be deſcribed in it. The price to ſubfubfcribers will be 120 livres, 24 of which are to be paid at the time of fubfcribing. The work will be publifhed in 12 numbers. The firft is to appear in June 1781, and the whole impreffion is to be completed in two years.

---

The fection of the fymphyfis pubis has lately been performed with fuccefs in Spain, by Signor Canivell, furgeon major to the Naval Hofpital at Cadiz. The patient, a lady aged forty-two years, was taken in labour of her firft child on the 6th of Auguft laft. The labour pains continued during twenty-four hours, but the head of the fœtus being prevented from advancing by the narrownefs and diftortion of the pelvis, and the patient being much reduced, her accoucheur thought the operation adviseable. It was accordingly performed without wounding the integuments. The incifion was begun above the clitoris, and the operator was careful to injure the meatus urinarius as little as poffible. As foon as the feparation was effected, a crepitus was heard, and in a few minutes the patient was delivered of a live child. The fever foon went off, and on the 38th day fhe got out of bed. The only inconvenience fhe feels at prefent, is a flight incontinence of urine.

The

---

The Academy of Sciences at Bourdeaux having offered a prize of 450 livres for the beſt paper on the incontinence of urine, to which many perſons are ſubject when in bed, and on the means of curing it, M. Leger, a ſurgeon at Paris, has, without any view to the prize, communicated to the public a remedy, by means of which he has ſucceeded in three caſes of this ſort. It conſiſts of ſix grains of cantharides in powder, mixed with two drachms of extract of borage, and divided into 24 doſes, one of which is to be taken every night at bed-time. In one of the patients, a young lady, 24 years old, who had been ſubject to the above-mentioned complaint from her infancy, the medicine was repeated 72 nights, and the doſe of cantharides increaſed a few grains. This patient was perf[illegible]ly cured, and for two years has enjoyed the beſt health. The two other patients were ſiſters, the eldeſt fifteen and the youngeſt thirteen years old. The remedy produced in both the ſame good effect as in the caſe juſt now mentioned, and they have now been well a year. By way of precaution, they were all directed to drink linſeed tea, but the medicine occaſioned no ſtrangury in either of them.

---

On the 9th of December 1780, the College of Physicians at Paris held their Anniversary Meeting in the schools of the Sorbonne. On this occasion were delivered the Eulogies of Doctors Joseph de Jussieu, Albert Hazon, and William Michel, three members of the college, who died in the course of the preceding year. M. Destandes read a dissertation condemning the use of opium in intermittents, previous to the paroxysm, a practice introduced about thirty years ago by M. Berryat, physician at Auxerre. M. Mallet gave an account of a new species of bark brought from Martinico, and which seems to be the same as that lately described in the *Phil. Trans.* by Dr. Wright. M. Majault communicated some observations on vinegar, proving it not to be an antidote to arsenic, as some have lately asserted.

---

A second volume of Experiments and Observations relating to various branches of Natural Philosophy, &c. by the celebrated Dr. Priestly, is in the press, and will speedily be published. We are likewise informed that the Abbé Fontana is printing a very interesting work on Poisons.

The

The Memoirs of the Academy of Sciences at Berlin, for 1779, lately publiſhed, contain ſome account of the late Dr. J. H. Pott; this celebrated Chemiſt was born at Halberſtadt in 1692, and graduated at Halle in Saxony. His inaugural diſcourſe was on the ſulphur of metals. In 1718 he was admitted of the Academy, and about the ſame time was appointed Profeſſor of the Theory of Chemiſtry in the College of Phyſic and Surgery at Berlin. He died on the 29th of March 1777, aged eighty-five years, leaving ſeveral daughters. His *Lithogeognoſia* and other writings are well-known to the chemical reader.

